# Facial Nerve Palsy as Complication in COVID-19 Associated Mucormycosis: A Case Series

**DOI:** 10.7759/cureus.33077

**Published:** 2022-12-29

**Authors:** Prashanthi Reddy, Ajay Parihar, Renu Singh, Varsha AC, Ajay Sutare

**Affiliations:** 1 Department of Oral Medicine and Radiology, Government College of Dentistry, Indore, IND

**Keywords:** covid-19, diabetes mellitus, rcm, mucormycosis, facial nerve palsy

## Abstract

Mucormycosis is an opportunistic fungal infection indicating a high mortality rate. Among six varieties of involved sites, rhino cerebral mucormycosis (RCM) is not the most uncommon. During the COVID-19 pandemic, with the increase, in predisposing conditions incidence rate of mucormycosis progressed. For aggressive treatment, an early diagnosis can be armored to reduce morbidity and mortality. Clinically RCM poses non-specific symptoms and signs delaying diagnosis. This is associated with orbital cellulitis and sinusitis, one-sided headache behind the eye, diplopia, blurring of visions, nasal congestion, rhinorrhea, epistaxis, nasal hypoesthesia, facial pain and numbness, and a history of black nasal discharge. Not commonly the complications of cranial nerve involvement have been reported. In the present case series, three presentations of facial nerve palsy in COVID-19 associated with mucormycosis are added to the literature database.

## Introduction

During the current pandemic of COVID-19 (Coronavirus disease 2019), a large number of manifestations and complications have emerged including fungal infections, and are being reported in the literature. Lately, fungal infections are emerging complications as a matter of concern in patients diagnosed positive for COVID-19. Mucormycosis is an opportunistic fungal infection with a high mortality rate caused by saprophytic fungi (Phycomycetes, zygomycetes, mucoraceae). Rhizopus oryzae is the most common fungi isolated from patients with mucormycosis and is responsible for 70% of all cases [1]. It is frequently found in soil, residue plants, spoiled food, and the upper respiratory tract of the healthy host. It becomes pathogenic when predisposing factors cause an immunocompromised state with a decreased ability to phagocytize, most commonly observed in diabetes (60-80%), hematological neoplasms, chemotherapy-induced neutropenia, and the use of deferoxamine therapy. The incidence rate of mucormycosis varies from 0.005 to 1.7 per million populations and the case fatality rate is 46% [2]. On the basis of site of involvement, the disease may be classified into six different forms, rhino cerebral, pulmonary, cutaneous, gastro-intestinal, orbital, and disseminated of which rhino cerebral mucormycosis (RCM) is the commonest type accounting for about 30-50% of cases [3]. Clinical presentation of RCM includes nasal stiffness, headache, retro-orbital pain, orbital swelling, and rarely facial palsy. Early diagnosis of RCM is difficult. The incidence of facial nerve palsy in uncontrolled diabetic patients with mucormycosis is 11% [4].

Also, several neurological syndromes like anosmia/ageusia, encephalitis, encephalopathy, cerebrovascular complications, myelitis, Guillain-Barré syndrome, and facial palsy are among the neurological complications manifested in a significant proportion in COVID-19 [5]. Facial palsy is reported in the literature, during the clinical course of the infection or as its first symptom in these patients [6,7]. The present case series intends to raise awareness of this unusual presentation of facial nerve palsy in RCM. Additionally, it pretends to explore the pathophysiology of facial nerve palsy in mucormycosis.

## Case presentation

Case 1

**Figure 1:**
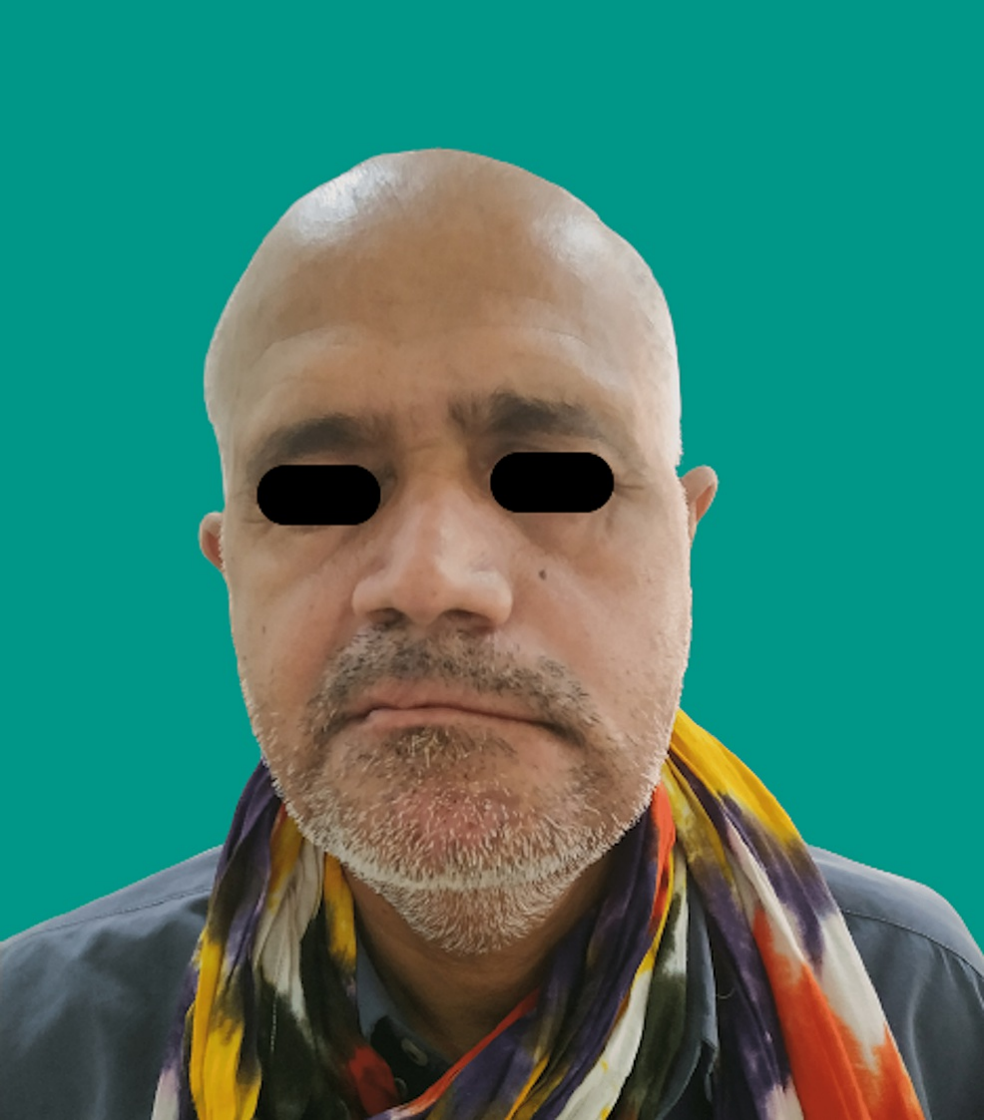
Unilateral House Brackman grade V facial nerve palsy involving the left side.

A 48-year-old man presented with the chief complaint of swelling on the left side of his face for 20 days. Anamnestic, the patient was hospitalized for 15 days after reverse transcription-polymerase chain reaction (RT-PCR) tested positive for COVID-19 and was found to have an elevated blood glucose level of 351mg/dl during treatment. Extraoral examination revealed facial asymmetry and facial palsy involving the left side of the face were present. Clinical features of facial palsy included drooping of the corner of the mouth, the absence of wrinkles in the right half of the forehead, and incomplete closure of the right eye with a barely perceptible motion of the left side of the face. As a result, it appears that the patient may have House Brackman grade V unilateral facial nerve palsy (Figure 1).

**Figure 2:**
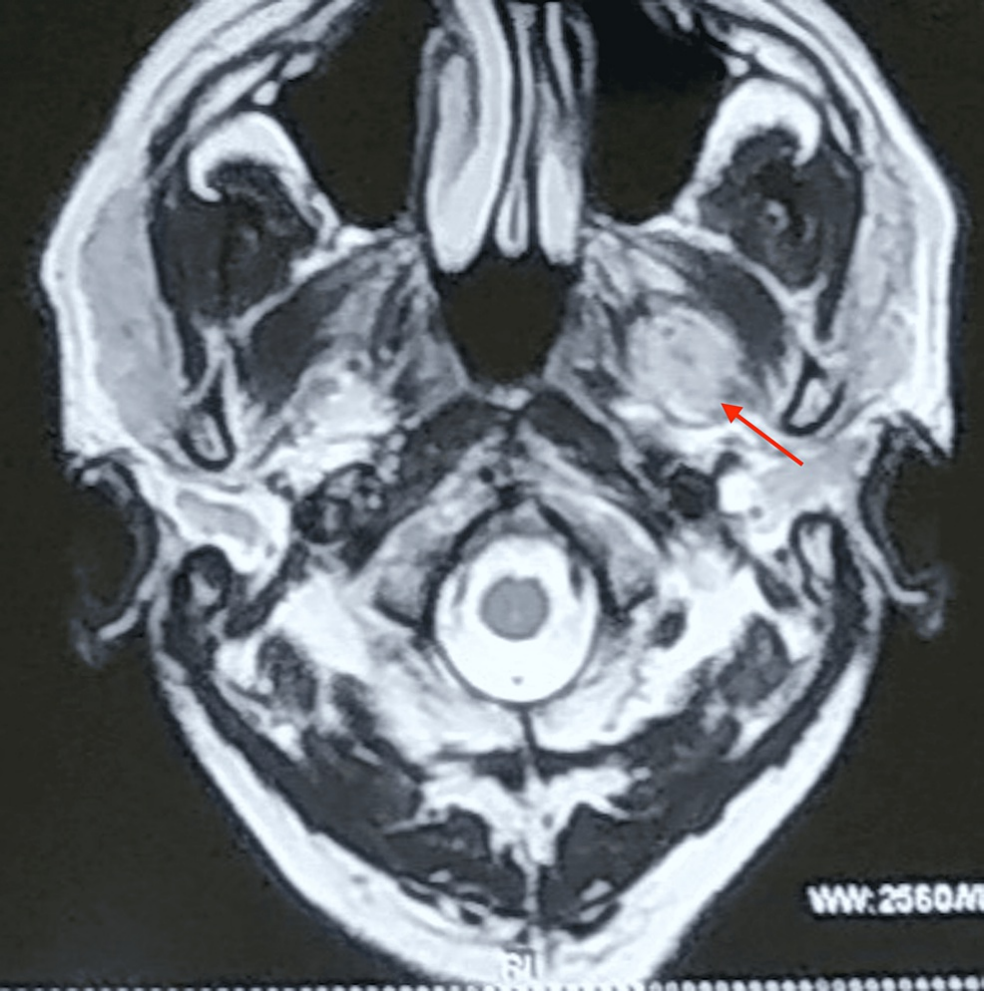
MRI T2 axial section shows hyperintense, peripherally enhanced collections in the deep intramuscular plane of the bilateral pterygoid fossa. MRI: magnetic resonance imaging

Intraorally, necrotized bone was noted in the mandibular anterior teeth and left parasymphysis in association with mobility of 35, 36, and 47. The magnetic resonance imaging (MRI) report showed some poorly defined, T2 hyperintense, peripherally enhanced collections in the deep intramuscular plane of the bilateral pterygoid fossa, extending into the bilateral parapharyngeal space, associated with mild surrounding soft tissue strands suggestive of abscess formation (Figure 2). The final diagnosis of mucormycosis of the pterygoid fossa with extension into the parapharyngeal space was made on the basis of histologic examination.

Case 2

**Figure 3:**
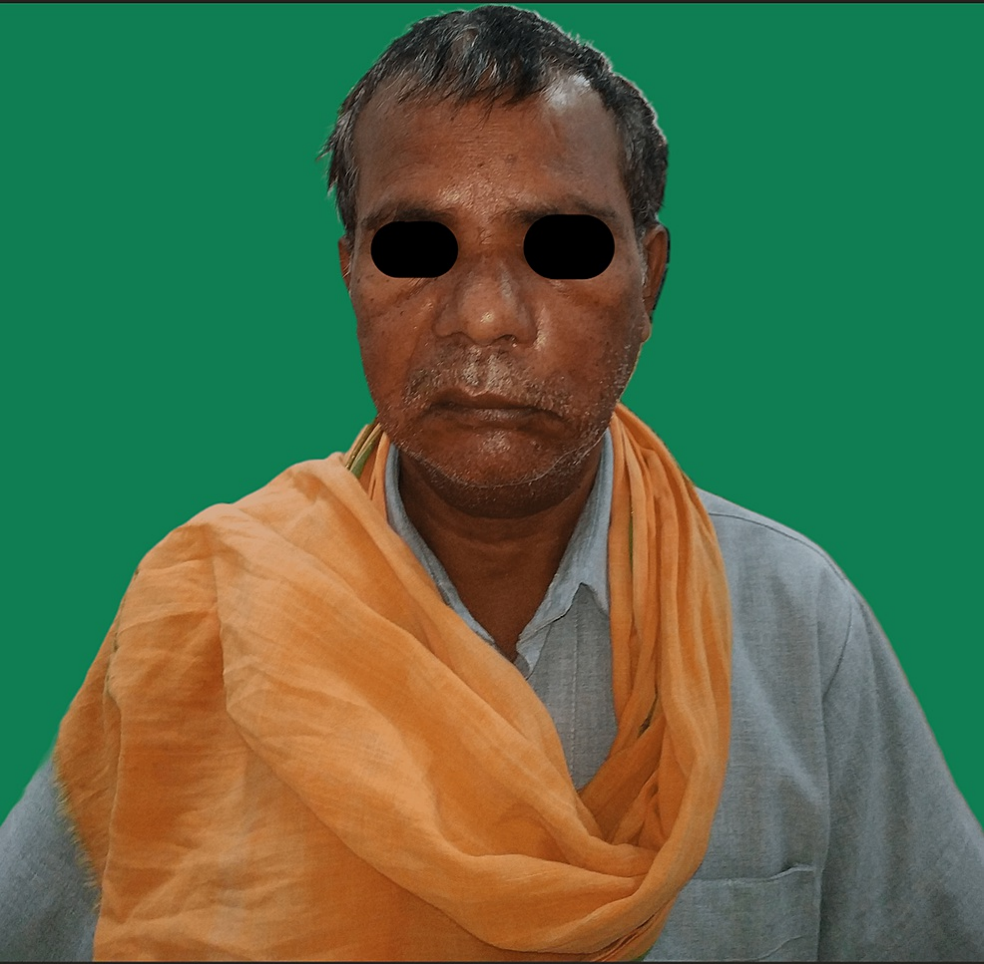
Unilateral House Brackman grade V facial nerve palsy on the left side.

A 50-year-old man presented clinically with swelling on the left side of his face and blackish discharge from the left nasal cavity for 10 days. A history of diabetes mellitus was diagnosed during the treatment of COVID-19. Extraoral examination revealed facial asymmetry, unilateral House Brackman grade V facial nerve palsy on the left side, and tenderness in the left infraorbital and zygomatic regions (Figure 3). Features of facial palsy seen were drooping of the corner of the mouth, absence of wrinkles on the right half of the forehead, and incomplete closure of the right eye clinically confirming House Brackman grade V unilateral facial nerve palsy.

**Figure 4:**
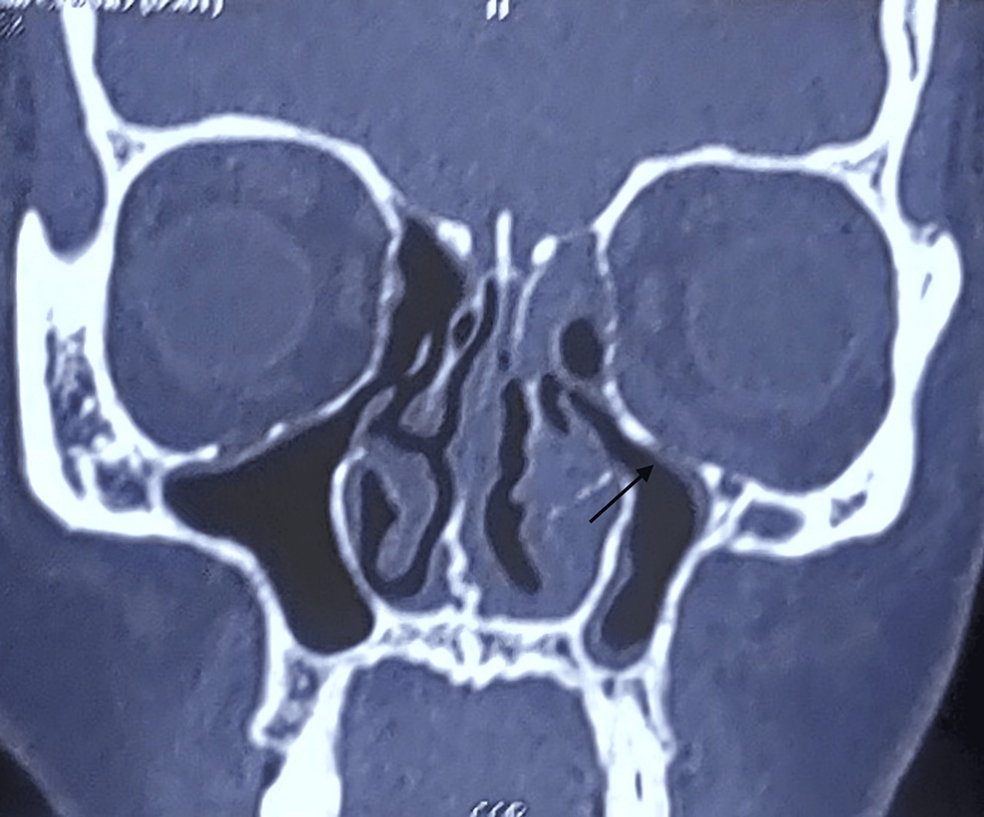
CT PNS reveals mild mucosal thickening of the left paranasal sinus, alteration in the shape of the left inferior turbinate, mid-nasal septal deviation, and break in the continuity of the medial wall and floor of the orbit. Arrow showing break in continuity of orbital wall. CT PNS: computerized tomography of the paranasal sinus

Computerized tomography of the paranasal sinus (CT PNS) findings suggested acute fungal sinusitis with pre and paraseptal orbital cellulitis (Figure 4).

**Figure 5:**
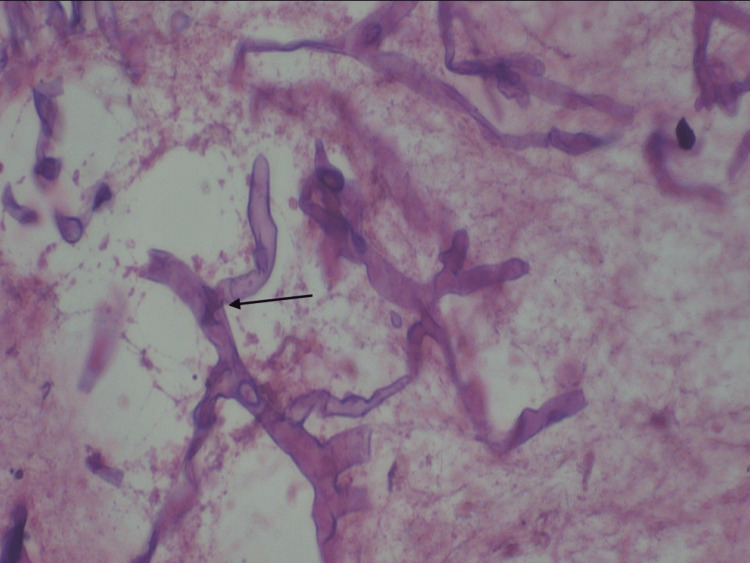
H&E stained section shows mucosa infiltrated by broad non-septate hyphae branching at right angles. H&E: hematoxylin & eosin

The final diagnosis of mucormycosis of the rhino-orbital region was made on the basis of histologic examination. On histopathological examination of hematoxylin & eosin (H&E) stained sections shows sino-nasal mucosa infiltrated by broad non-septate hyphae branching at right angles along with mixed inflammatory cell infiltrate (Figure 5). The patient was subjected to endoscopy-assisted bilateral nasal cavity debridement under general anesthesia along with anti-fungal therapy.

Case 3

**Figure 6:**
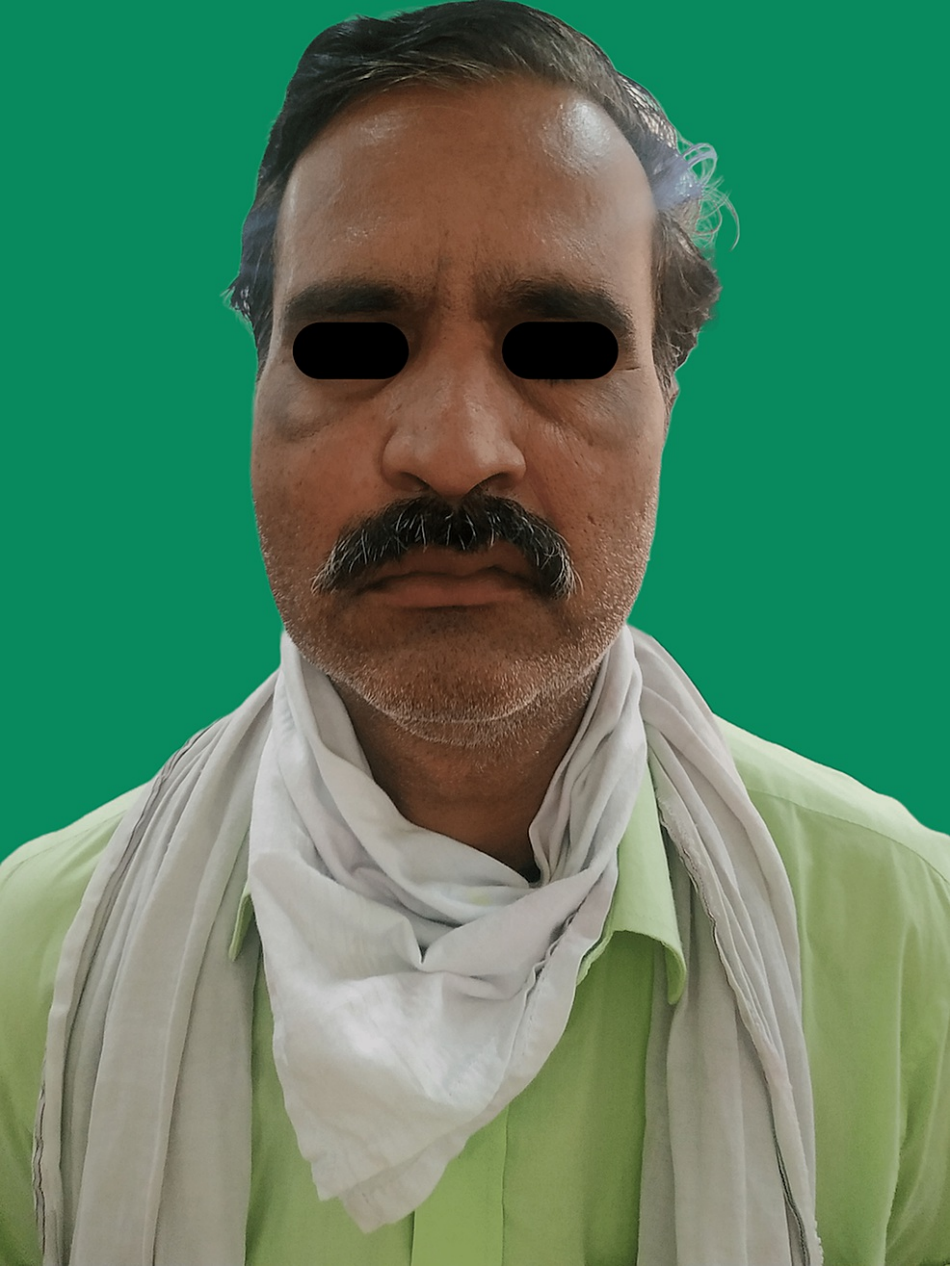
House Brackman grade IV unilateral facial nerve palsy.

A 43-year-old male patient presented with the main concern of having swelling on the right side of his face for one month. His medical history included controlled diabetes mellitus for one year and recent hospitalization for COVID-19 infection. Extraoral clinical examination revealed facial asymmetry and features of unilateral facial nerve palsy according to House Brackman (IV) (Figure 6).

**Figure 7:**
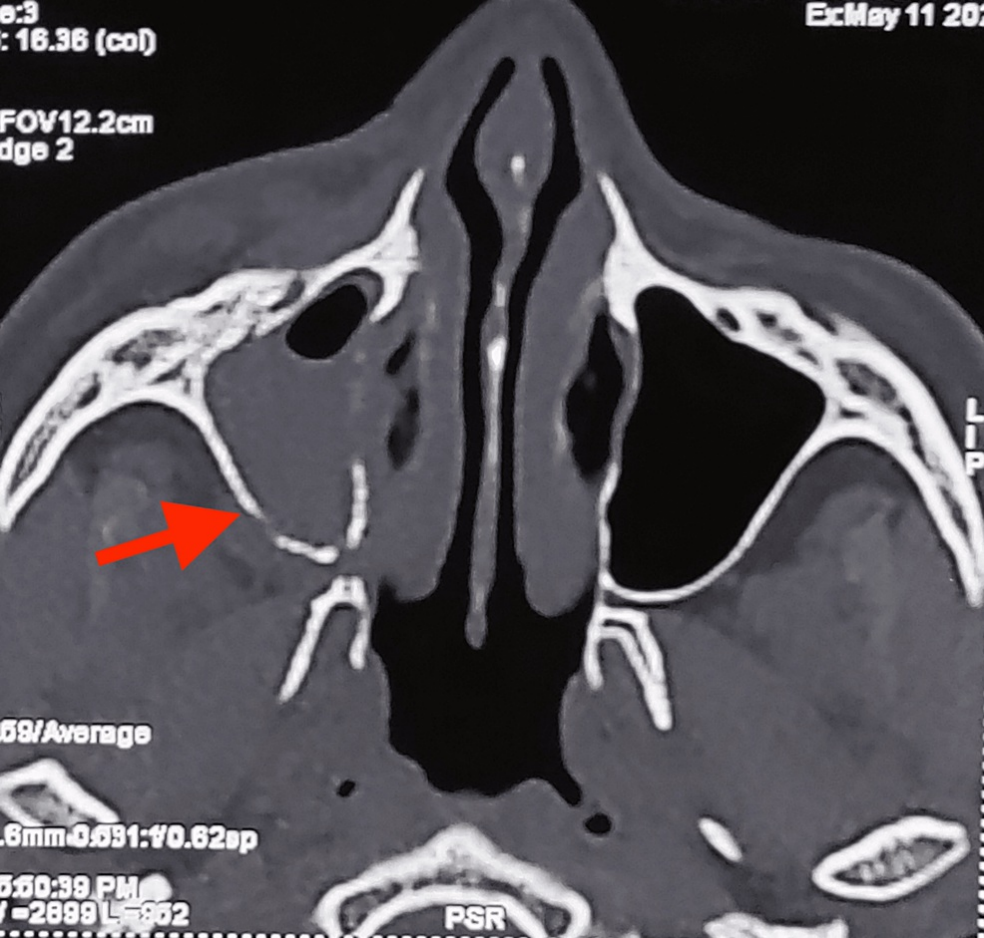
CT PNS axial section showing sinusitis of the right maxillary sinus with pervasive erosion of the entire maxillary sinus wall. CT PNS: computerized tomography of the paranasal sinus

Palpation of the right malar region revealed tenderness. Intraorally, the right upper first molar showed grade 1 mobility. Examination of CT PNS revealed increased mucosal thickening within the right maxillary sinus representing maxillary sinusitis of the right maxillary sinus with pervasive erosion of the entire maxillary sinus wall of the right maxillary sinus (Figure 7).

**Figure 8:**
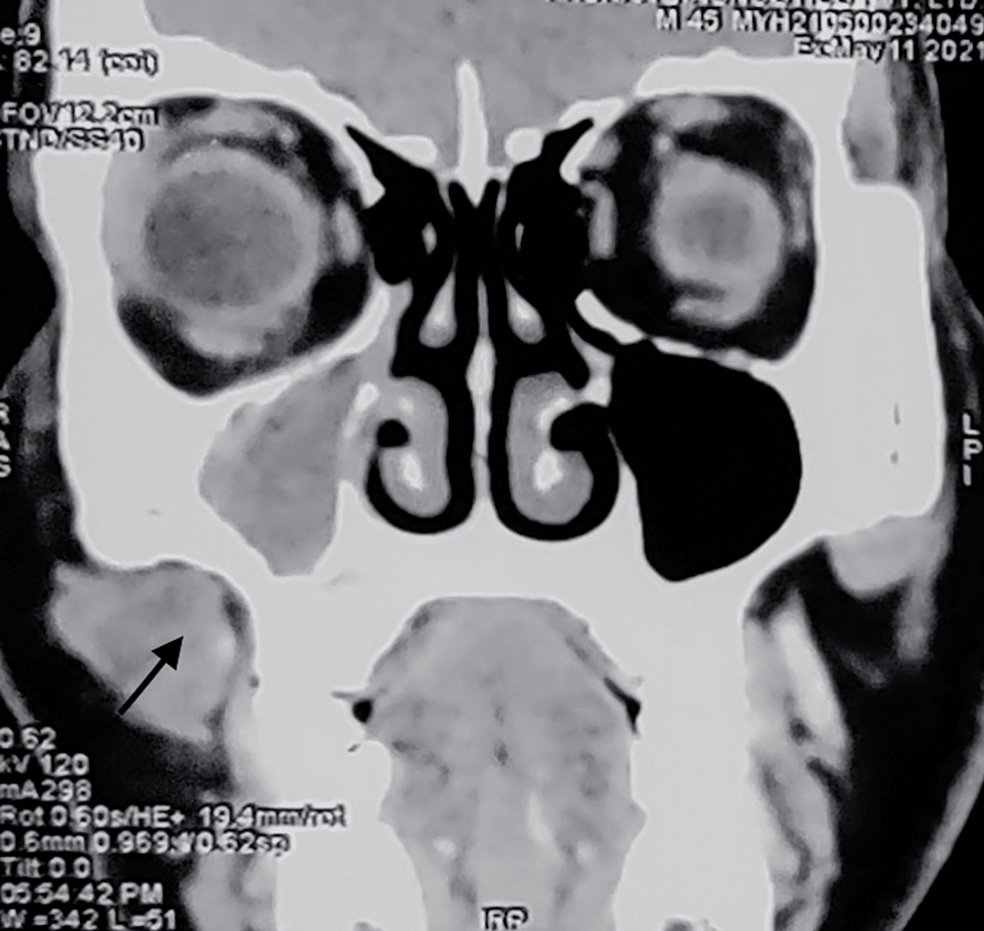
CT coronal section showing mildly enhancing soft tissue in the pre-maxillary region. CT: computed tomography

Also, ill-defined mild-enhancing soft tissue extending from the pre-maxillary region to the right parotid space along the parotid duct and the neurovascular bundle was noted (Figure 8).

**Figure 9:**
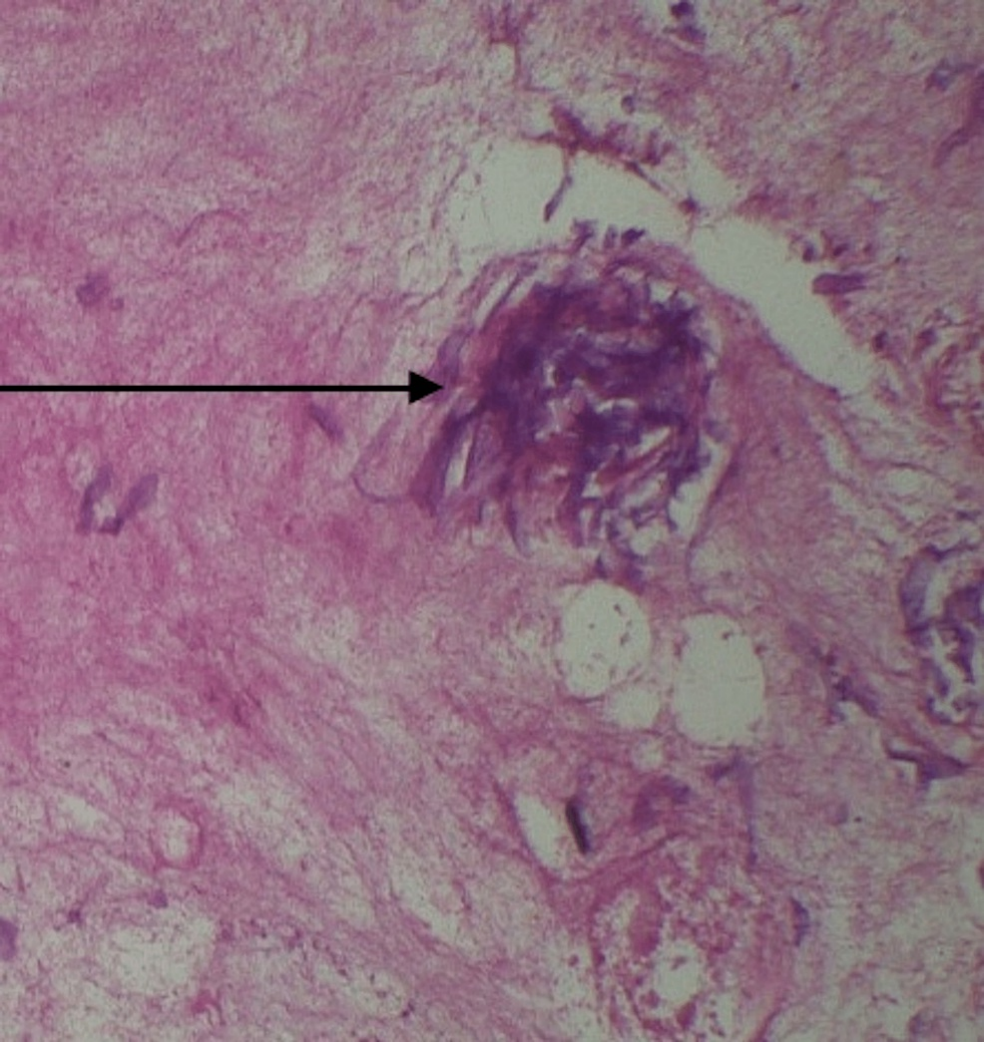
H&E stained section (10x) shows aggregates of hyphae within the vasculatures. H&E: hematoxylin & eosin

The patient underwent right maxillary sinus debridement with right side inferior alveolectomy under conscious sedation, right eye tarsorrhaphy, and functional endoscopic sinus surgery. The patient was under Amphotericin B (liposomal) 10mg/kg daily, posaconazole 300mg in addition to ceftriaxone, with metformin 500mg, glimepiride 2mg, and pioglitazone 1.5mg 1/2 BD. The histopathology report was suggestive of mucormycosis. In H&E stained section moderate degree of tissue invasion with the fungus was noted. The fungal invasion was characterized by broad, short, aseptate, obtuse branching and eosinophilic hyphae at multiple sites mostly confined with the necrotic tissue and vessels (Figure 9).

## Discussion

It is not unusual for Rhizopusoryzae to be isolated from mucormycosis patients. In 70% of cases, it is to blame for mucormycosis. Patients with COVID-19 with uncontrolled diabetes mellitus have seen an increase in the number of mucormycosis cases recently [8]. COVID-19, an emergency global public health event, caused by severe acute respiratory syndrome coronavirus 2 (SARS CoV-2) presented as diffuse alveolar damage and severe inflammatory exudation with an immunosuppression state. Factors that facilitate fungal infection in patients with COVID-19 may include low oxygen (hypoxia), high glucose (diabetes, new-onset hyperglycemia, steroid-induced hyperglycemia), acidic medium (metabolic acidosis, diabetic ketoacidosis (DKA)), high iron levels (increased ferritins) and decreased phagocytic activity due to immunosuppression (SARS-CoV-2 mediated, steroid-mediated or background comorbidities), and several other shared risk factors including prolonged hospitalization with or without mechanical ventilators [9].

Early diagnosis of RCM clinically is difficult. Frequent clinical presentation includes malaise, headache, facial pain, swelling, and mild fever. The involvement of various tissues in the rhino-cerebral area can lead to clinical presentation mimicking other conditions like cerebrovascular accidents (CVA) [10]. Facial nerve palsy as a presentation of RCM has also been reported in a few isolated cases. The exact pathology of the involvement of the facial nerve is unknown. The involvement of pterygopalatine fossa has been reported by some authors as a route for the spread of mucormycosis to the facial nerve. The pterygopalatine fossa is also considered to be a reservoir of mucor from where it spreads to retro global space of orbit and infratemporal space [11]. Recent studies have demonstrated the spread of Mucorales species along peripheral nerves [12]. Another reason for the involvement of facial nerve can be the pathology of resistance arteries in diabetic patients which may cause edema and localize facial nerve ischemia. This would compromise the blood supply to the nerve leading to palsy [13].

Diabetes mellitus is itself an immunocompromised stage, changing the normal immunological response to infection in several ways. A hyperglycemic state stimulates the proliferation of fungus and also decreases chemotaxis and phagocytic efficiency permitting the organism to grow well in the environment. Enzyme ketoreductase is produced by the fungus Rhizopus oryzae, which allows them to utilize ketone bodies in diabetic ketoacidosis patients, and result proliferation of pathogens [14]. And also, the subclinical facial nerve is involved in 6% of the patients with diabetes reported [15]. In a study series of 126 patients with Bell's palsy, chemical or overt diabetes mellitus was found in 39% of the cases. In the same study disturbances of taste were found more in non-diabetic patients (83%), as compared to only 14% of diabetic patients whose taste was affected. Thus, a common site of facial nerve lesions in diabetics appears to be distal to the chorda tympani. This may only be explained by diabetes-related pathogenesis and a vascular rather than a generalized "metabolic" impairment, leading to localized facial nerve ischemia in the distal part of the fallopian canal. Thus, some cases of Bell's palsy may be due to diabetic mononeuropathy [13]. Mucormycosis and hyperglycemia have been considered as the pathophysiology for facial paralysis in the three cases that have been documented.

Mucormycosis and hyperglycemia have been considered as the pathophysiology for facial paralysis in the three cases that have been documented. Recent studies also suggested a neuroinvasive capacity of COVID-19 and facial palsy in COVID-19 patients as initial findings, during the course of treatment or after recovery. According to studies this virus has a high affinity for angiotensin-converting enzyme-2 (ACE-2) receptors, which are frequently found in the nervous system, it performs neurotropism by directly causing nerve damage [16]. Dubé et al. reported in their study with animal models that there is axonal transport of human coronavirus (HCoV) OC43 protein into the nervous system [17].

Antifungal therapy, surgical debridement, supportive therapy, and prosthetic devices are all used in the course of treatment. We provide a case series of three mucormycosis cases that were identified based on clinical findings, radiographic findings, and laboratory testing and presented to the outpatient clinic. Patients in every case experienced mucormycosis following recovery from COVID-19 disease. Squamous cell carcinoma, chronic granulomatous infections including tuberculosis, midline lethal granuloma, rhinosporidiosis, tertiary syphilis, and other deep fungal infections should all be considered in the clinical differential diagnosis of the lesion.

All three individuals show COVID-19 infection, high blood sugar, and histopathologic evidence of mucormycosis. The pterygoid fossa is thought to be the starting place for the spread of infection, hence the MRI data from case 1 implies that the pterygoid fossa may be involved, which may be the cause of the facial paralysis. In case 3, the CT PNS scan reveals right parotid gland involvement along the neurovascular bundle, which may also be a contributing factor to facial palsy.

## Conclusions

In a few rare cases, mucormycosis has been linked to facial nerve palsy. Contrast-enhanced MRI can show the extent of mucormycosis. The expansion of the pterygopalatine fossa, which is discernible on an MRI, can be used to identify facial nerve involvement. This case series is a contribution to the literature database, but it is still unclear how facial nerve palsy affects individuals with mucormycosis as a whole. Facial nerve palsy must be taken into account as a clinical consequence to improve the quality of life for individuals with mucormycosis.
